# Effect of Density of Acrylic Acid Ester on Sulfonate-Modified Polycarboxylate Superplasticizers on Cementitious Systems

**DOI:** 10.3390/polym16233272

**Published:** 2024-11-24

**Authors:** Yuxiang Xie, Zixuan Zhang, Yujie Chen, Xu Ren, Yuan Liu, Jia Tao, Runxia Liu, Min Li, Ziwei Li

**Affiliations:** 1 Guizhou Provincial Key Laboratory of Green Chemical and Clean Energy Technology, School of Chemistry and Chemical Engineering, Guizhou University, Guiyang 550025, China; 13899569538@163.com (Y.X.); 18834163770@163.com (Y.C.); renxu1256@163.com (X.R.); minli@git.edu.cn (M.L.); 2 North Alabama International College of Engineering and Technology, Guizhou University, Guiyang 550025, China; chunmanyueyuan@163.com; 3 School of Civil Engineering, Guizhou Institute of Technology, Guiyang 550025, China; 4 China Railway Fifth Bureau Group Co., Ltd., Guiyang 550003, China; xyx2277466012@163.com (Y.L.); xyx122079@163.com (J.T.); toudiyong123@163.com (R.L.); 5 Guizhou Tianwei Building Materials Technology Co., Ltd., Guiyang 550025, China

**Keywords:** polycarboxylate superplasticizer, conformation, anion density, adsorption capacity, viscosity reduction

## Abstract

To tackle high viscosity in fresh concretes, especially high-performance concrete, the research of polycarboxylate superplasticizers (PCEs) is relevant. By designing the molecular structure of PCEs, problems such as pumping difficulties in high viscosity of high-performance concrete can be solved. Therefore, in this paper, a suite of novel viscosity reducing PCEs containing sulfonic acid groups and different acrylate densities were synthesized on the basis of inventive molecular structure design, and characterized to determine the predicted structure. The maximum adsorption, the best fluidity, and the Minimum zeta potential value can be seen for PCEs with a small number of ester groups (PCE-MA0.5) due to the combination of the rigidity of its backbone and the density of the adsorption groups. Moreover, the investigation of working mechanism showed the introduction of ester groups can significantly reduce viscosity, but also reduces the adsorption capacity. This research aims to propose a feasible method for synthesizing PCE with superior processability and viscosity reduction capability in cement and concrete.

## 1. Introduction

The progressive evolution of the construction sector necessitates enhanced durability and strength in concrete, notably in large structures, hence the profound application of high-performance concrete (HPC) and ultra-high performance concrete (UHPC) [1,2,3]. However, existing strategies to improve strength of concrete are mainly through reducing the water cement ratio (w/c) and increasing amounts cementitious materials amounts [4,5]. These strategies will cause the viscosity of fresh mixed concrete to increase, the flow rate to slow down, incurring difficulties in subsequent transportation, construction, and pumping [6,7,8]. Polycarboxylate superplasticizers (PCE), as an admixture, are widely applied in concrete due to their high water reduction rate and good fluidity retention abilities at low dosages, which improve the workability of concrete [9,10,11]. PCE is a kind of comb-like polymers that preferentially adsorbs its backbones and forms a brush layer by adsorption [10]. Through an array of outgoing side chains, this layer can be used as an excellent colloidal stabilizer and lubricant [12]. At present, the main measures taken to solve the issue of excessive viscosity in concrete are increasing the dosage of PCEs, selecting high-quality ultrafine powder and optimizing particle size distribution [13,14,15]. However, selecting high quality ultrafine powder and optimizing particle grading are limited by the geographical location of cementitious materials and additional construction cost [16,17,18,19]. And high dosage of PCEs brings high construction cost, deferred setting, bleeding and segregation [20,21]. In addition, PCE is often combined with viscosity modifying admixture (VMA) to solve the problems of bleeding and segregation of concrete [22,23]. However, the admixture of VMA with PCE could induce compatibility problems within cement pore solution, causing competitive adsorption on cement particle surface, thus influencing their properties [22,24,25]. Therefore, developing PCEs with excellent viscosity reduction performance has become a research hotspot in the industry.

When it comes to designing PCE molecules, a variety of methods can be utilized. Due to PCEs comb-like structure, the molecular weight of comb-like polymers can be modified by tweaking the length of side chains [12,26], the density of the adsorption groups of the polymers can be controlled by the degree of polymerization of main chain [27], and the hydrophilic and hydrophobic properties of the polymers can be manipulated by monomers and functional groups [28]. Some researchers delved into the preparation of PCEs from the perspective of molecular structure. Qian et al. [29] synthesized a viscosity reducing PCE by reducing side chain length of PCEs and introducing hydrophobic groups. At the fixed w/c, the plastic viscosity of concrete after adding viscosity reducing PCE reduces by more than 40%. Ma et al. [30] assessed the impact of various adsorption groups on the performance of PCE. It was found that by introducing additional sulfonic acid groups to the PCE molecule, the synthesized S-PCE had least sensitivity to cement and the best fluidity. Zhang et al. [31] research demonstrated that as the acrylate segment in the main chain of PCE molecule increased, PCEs exhibited delayed dispersion, which helped to improve the dispersion retention of fresh concrete. This is due to the continuous hydrolysis of the acrylate segment in an alkaline environment, producing new R-COO^−^. Among above reports, it is evident that the introduction of hydrophobic groups has a positive influence on reducing the viscosity of concrete, and the existence of sulfonic acid groups helps to reduce sensitivity of PCEs to cement. Therefore, by designing the molecular structure of PCEs, a series of PCEs containing both sulphonic and ester groups have been synthesized, which have great potential for practical applications. Moreover, this paper presents a systematically analysis of the alterations in the molecular structure and performance of PCEs resulting from the incorporation of ester groups. This provides a valuable point of reference for the molecular design of PCEs.

In this paper, the anionic density, rigidity and hydrophobicity of PCEs were adjusted by introducing sulfonic acid group and different content of ester group. The effects of different ester content of PCEs on the fluidity and workability of cement and concrete were deliberated. Furthermore, the analytical evaluation of PCEs encompassed measurement of their surface tension in water and zeta potential post adsorption onto cement particles. The working mechanism of synthetic PCEs was explored derived from the discussion of PCEs workability and physicochemical properties, which put forward new interpretations on the development direction of PCEs.

## 2. Experimental

### 2.1. Materials

Methyl allyl alcohol polyoxyethylene ether (HPEG, M_w_~2400 g/mol) was obtained from Liaoning Kelong Fine Chemical Co., Ltd. (Liaoyang, China). The crude materials for polymer production such as acrylic acid (AA, AR), methyl acrylate (MA, AR), sodium methallyl sulfonate (SMAS, AR), sodium hydroxide (NaOH, AR), and ammonium persulfate (APS, AR) were all procured from Shanghai Aladdin Biochemical Technology Co., Ltd. (Shanghai, China).

For this research, the selected reference cement was elegantly complied with the standard GB 8076-2008 under surveillance of China Building Materials Academy, sourced from Fushun Aosaier Technology Co., Ltd. (Fushun, China). It has specific surface area of 356 m^2^/kg and specific density of 3.11 g/cm^3^. The chemical and mineral components of the reference cement are tabulated in Table 1.

### 2.2. Synthesis of the Polymers

**Figure 1:**
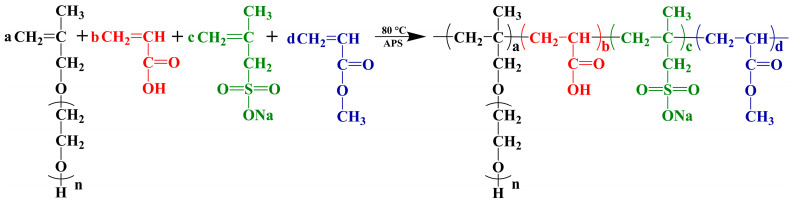
Synthesis route of PCEs with different ester group content.

By selecting different molar ratios of HPEG, AA, SMAS, and MA (Table 2), five PCEs with different ester group content had been synthesized via the route as shown in Figure 1. For instance, the procedure for preparing PCE-MA0.5 by radical polymerization of HPEG, AA, SMAS, and MA was as below. HPEG solid powder (36.25 g) and deionized (DI) water (30 mL) were incorporated into a three-neck flask with stirring for 20 min at 30 °C, followed by heating to 80 °C. The initiator solution (0.18 g APS, 0.11 g MPA, and 10 mL DI water) and the monomer solution (4.35 g AA, 0.65 g MA, 0.096 g SMAS, and 6 mL DI water) were blended in a three-neck flask within 1 h, followed by stirring for an additional hour. Finally, the pH of solution was regulated at 7 ± 1 via 30 wt.% NaOH aqueous solution.

### 2.3. Gel Permeation Chromatography (GPC)

Number-average molecular weight (M_n_), weight-average molecular weight (M_w_), and polydispersity index (PDI = M_w_/M_n_) were obtained from an Agilent 1260 Infinity II GPC (Santa Clara, CA, USA). The 1.0 mg/L samples were evaluated with 0.1 mol/L of NaNO_3_ eluent under 1.0 mL/min fluid velocity.

The conversion rate (*R_h_*) was calculated by the following formula [32].

(1) $$R_{h}\%=\left(1-\frac{1}{f}\times \frac{A_{1}}{A_{1}+A_{2}}\right)\times 100\%$$

where *A*_1_ and *A*_2_ denote the proportion of peak area occupied by HPEG and PCEs GPC spectra, respectively; *f* denotes the mass fraction of HPEG.

### 2.4. Fourier Transform Infrared (FTIR)

The infrared spectra of PCEs had been analyzed by a Nicolet iS50 FTIR spectrometer (Thermo Fisher Scientific, Waltham, USA) with a scanning range of 4000~400 cm^−1^. 10 μL of 10 wt.% PCE solutions dropped onto plates pressed by 100 mg KBr at 15,000 KPa. After that, the plate was fixed to the bracket and scanned 64 times. Before FTIR test, the spectrometer was calibrated to eliminate the impacts of background.

### 2.5. ^1^H Nuclear Magnetic Resonance (^1^H MMR)

^1^H NMR spectra of the PCEs were measured by a JNM-ECZ400S/L1 spectrometer (JEOL, Tokyo, Japan) working at a resonant frequency of 400 MHz and using 32 times scan. 10 mg of freeze-dried PCEs were dissolved in D_2_O (δ = 4.70 ppm) to prepare ^1^H NMR samples.

### 2.6. Dynamic Light Scattering (DLS) Measurements

The hydrodynamic radius (*R_h_*) of PCEs residing within the cement pore solution was determined via the dynamic light scattering method utilizing a Morven NanoZS-90 (model: ZEN3690, location: UK) light scatter instrument operating at 90° measurement angles. During a customary experimental procedure, cement and water are meticulously incorporated into a blender at 0.29 w/c and agitated at 62 rpm for a duration of 5 min. After that, the freshly blended slurry underwent 5 min of high speed centrifugation (10,000 rpm). The resultant supernatant liquid was separated from its constituents via a 0.45 μm filter. Subsequently, PCEs solid powders were dissolved into the above supernatant and made into 1.0 g/L. In the end, the DLS of the synthesized PCEs were measured using a cumulative analyzer.

### 2.7. Zeta Potential

Zeta potentials of varied PCEs were severally determined on a Delsa^TM^ Nano C analyzer (Beckman Coulter, Brea, CA, USA). In a typical test, different dosages of PCEs, 0–1.0% by weight of cement (bwoc), were dissolved in 200 g DI water. And then, 0.25 g reference cement (w/c of 800) was added in the PCEs aqueous solution. Each mixture was agitated for 3 min prior to analysis. Measurements were typically repeated 3 times and averaged as the final zeta potential value.

### 2.8. Surface Tension

Surface tension measurements were conducted on PCEs aqueous solutions applying an OSA60 analyzer (Ningbo NB Scientific Instruments Co., Ltd., Ningbo, China) with an operational measuring range between 0.01–2000 mN/m and a precision of ±0.01 mN/m. Three replications were performed and average values were derived to provide final figures.

### 2.9. Adsorption Amount on Cement

The adsorption capacity of PCEs on cement was assessed using a TOC-LCPH total organic carbon analyzer (manufactured by Shimadzu, Kyoto, Japan) in accordance with the depletion method [33]. Solutions (87 g) with different PCEs (bwoc = 0.12%) were prepared in advance. 300 g cement was mixed with 87 g PCEs solution on a cement pasta stirrer. After mixing for different durations (10, 30, 60, 90, and 120 min), an appropriate amount of cement pasta was taken out separately. Centrifugation of resultant pastes at 10,000 rpm for 5 min yielded supernatants for subsequent 50 times dilution in DI water to match the 100 mg/L calibration used in TOC analyzer.

The adsorption amount of PCEs onto cement was determined utilizing the formula provided [34,35,36]:

(2) $$\Gamma =\frac{(C_{0}-C_{t})\times V}{m}$$

where *Γ* stands for the adsorption amount of PCEs (mg/g); *C*_0_ refers to the initial concentration of the PCEs (mg/L); *C_t_* means the concentration of the PCEs at *t* min (mg/L); *V* signifies the volume of the solutions (L); *m* embodies the mass of cement (g).

The TOC of the pure pore solutions, resulting from minimal quantities of carbon derived of organic grinding aids and raw materials, was taken into account prior to the calculation of adsorption amounts of PCEs.

The pseudo-first-order and pseudo-second-order models can elucidate adsorption kinetics, as demonstrated by the following equations [35].

Pseudo-first-order model:

(3) $$\frac{1}{\Gamma }=\frac{K_{1}}{\Gamma_{\infty }t}+\frac{1}{\Gamma_{\infty }}$$

Pseudo-second-order model:

(4) $$\frac{t}{\Gamma }=\frac{1}{K_{2}\Gamma_{\infty }^{2}}+\frac{t}{\Gamma_{\infty }}$$

where $\Gamma_{\infty }$ denotes the saturated adsorption amount of PCEs (mg/g); *K*_1_ stands for the rate constant of the pseudo-first-order model (min^−1^); *K*_2_ refers to the rate constant of the pseudo-second-order model (g/(mg·min)).

### 2.10. Fluidity Examination of Cement Slurry

The initial fluidity and fluidity retention of cement slurries with 0.29 w/c were conducted at 25 ± 1 °C, in consonance with the Chinese standard method GB/T 8077-2012 [37]. In a conventional trial for initial fluidity, the cement slurry was formulated at 0.29 w/c and blended with PCEs whose concentrations ranged from 0.08 to 0.2% bwoc. The blend was slowly agitated for 120 s at 62 rpm, then allowed a reprieve of 15 s, concluded with a quick stirring for an additional 120 s at 125 rpm. After that, the stirred cement slurry was instantaneously deposited into a truncated cone with dimensions of 6.0 cm (base diameter), 3.6 cm (superior diameter), and 6.0 cm (height), positioned on a humectant glass plate, and then swiftly lifted. Subsequent to 30 s, the dispersion diameter of the cement slurry was assessed thrice, producing an average to represent the final spread value. For the analysis of fluidity retention, the cement slurry was stirred for 60 s at 125 rpm and was measured based on the above method. It was repeated every 30 min for 2 h.

### 2.11. Isothermal Calorimetry

Utilizing an isothermal conductivity calorimeter (TAM air, Thermometric, Stockholm, Sweden), 5 g cement, 1.75 g DI water (w/c = 0.35), and 0.2% PCEs (bwoc) were blended and poured into the calorimeter for 72 h to ascertain the hydration heat of the cement pastes. In addition, a blank sample without PCEs underwent identical testing.

### 2.12. Setting Time Test

According to the Chinese standard method GB/T 1346–2011 [38], the initial setting time and final setting time of cement paste were measured by Vicat needle method. The cement pastes were prepared utilizing procedure outlined in Section 2.10, except for 0.27 w/c and a fixed PCEs dosage of 0.12% (bwoc). The mixed cement pastes underwent curing at 20 °C and above 90% relative humidity. The initial setting time was recorded when the Vicat needle reached the depth of 4 ± 1 mm from the base. The final setting time appeared when Vicat needle penetrated into the cement paste 0.5 mm, and failed to produce a circular indentation on the surface.

### 2.13. Water Film Thickness (WFT)

Cement packing density was gauged through the wet packing method [38,39]. In a standard test, the mixed cement paste was inserted into a 50 mm × 50 cm cylindrical mold and weighed. The packaging density was computed utilizing the following equation [39,40,41].

(5) $$\emptyset =\frac{M/V}{u_{w}\rho_{w}+R_{c}\rho_{c}}$$

where Ø denotes packing density; *V* symbolizes mold volume. *M* signifies the mass of cement pastes; *R_c_* is the volume fraction of cement; *u_w_* refers to the volume fraction. *ρ_w_* and *ρ_c_* represent the densities of cement and water, respectively.

The WFT was determined via the equations as below [39,40,41].

(6) $$V_{ew}=V_{w}-\frac{1-\emptyset }{\emptyset }$$

(7) $$WFT=\frac{V_{ew}}{A}$$

where *V_ew_* represents the excess water ratio; *V_w_* represents the volumetric water-cement ratio; *A* is the total surface area of the cement.

### 2.14. Rheological Tests of Cement Paste

The rheology test was conducted with an advanced rotational rheometer (ARES-G2, New Castle, TA, USA). The cement pastes were prepared using the method described in Section 2.10. To minimize the influence of temperature, the cylinder and cement pastes were both sustained at 20 °C during the whole testing process. The shear rate was set as follow: (a) ascends linearly from 0 s^−1^ to 50 s^−1^ in 60 s; (b) maintains at 50 s^−1^ over 60 s; (c) decreases linearly from 50 s^−1^ to 0 s^−1^ in 60 s. The rheology parameters were attained form the linear reduction stage.

In this study, the down curve of shear stress versus shear rate was fitted by the Herschel-Bulkley (H-B) mode [30,42].

(8) $$\tau =\tau_{0}+K{\dot{\gamma }}^{n}$$

where *τ*_0_ denotes yield stress (Pa), *K* refers to the consistency index (Pa·s^n^), and *n* embodies the rheological index.

The equivalent plastic viscosity *μ* can be calculated from the following equation [42,43]:

(9) $$\mu =\frac{3K}{n+2}{\dot{\gamma }}_{max}^{n-1}$$

where *μ* is equivalent plastic viscosity (Pa·s); ${\dot{\gamma }}_{max}$ stands for the maximum shear rate (s^−1^).

### 2.15. Compressive Strength

The compressive strength of cement mortar incorporating diverse PCEs was evaluated as per GB/T 17671-1999 [44]. 1.35 kg standard sand, 0.45 kg cement, DI water (w/c = 0.34), and 0.15% PCE (bwoc) were stirred and transferred to a rectangular prism mode (4 cm × 4 cm × 16 cm). The compressive strength of the samples was tested after curing for 3, 7, and 28 d at 20 °C and above 95% humidity.

### 2.16. Concrete Performance

The concrete performance was assessed in accordance with GB 8076-2008 Chinese standards [45]. The concrete mixing proportion was aligned with Chinese standard of JGJ/T 55-2011 [46], as reflected in Table 3. The doses of various PCEs (20% solid content) were rectified to regulate the targeted slump flow of concretes within the limit of 600 ± 20 mm.

## 3. Results and Discussion

### 3.1. Structure Characterization of the PCEs

#### 3.1.1. GPC

**Figure 2:**
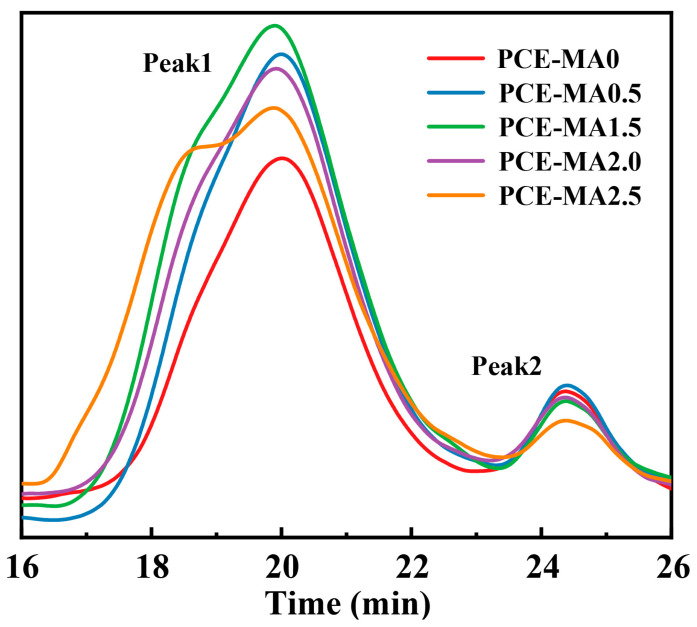
GPC spectra of the synthesized PCEs.

The M_w_, M_n_, PDI, and conversion rates of PCE samples were established via GPC, as detailed in Table 4 and Figure 2.

Depicted in Figure 2, there are two distinct peaks in the curves for all synthesized samples. Peak1 refers to the target molecules whose M_w_ are more than 45,000 g/mol. Peak2 corresponds to little amount of unreacted monomers with M_w_ around 2000 g/mol [47]. According to previous reports [48,49,50], excessively small molecular weight is detrimental to water-retaining property, and excessively large molecular weight harms cement paste dispersion. Thus, the M_w_ of PCEs molecule is acceptable, which is in the range of 47,281 to 74,872. The PDI in Table 4 solely describes the molecular weight distribution of polymers at Peak1. The PDI falls within a range from 1.99 to 2.95, indicating relatively uniform distribution of the synthesized PCEs molecular weight. If PDI is too large, there are too many products with too large molecular weight and too small molecular weight in the product, which is not conducive to the water-retention property and fluidity of cement containing PCEs. In addition, the conversion rate of the PCEs ranges from 81.9% to 92.4%, which means the occurrence of copolymerization reaction with high conversion rate and few unreacted monomers in the solution after the reaction.

#### 3.1.2. FT-IR

**Figure 3:**
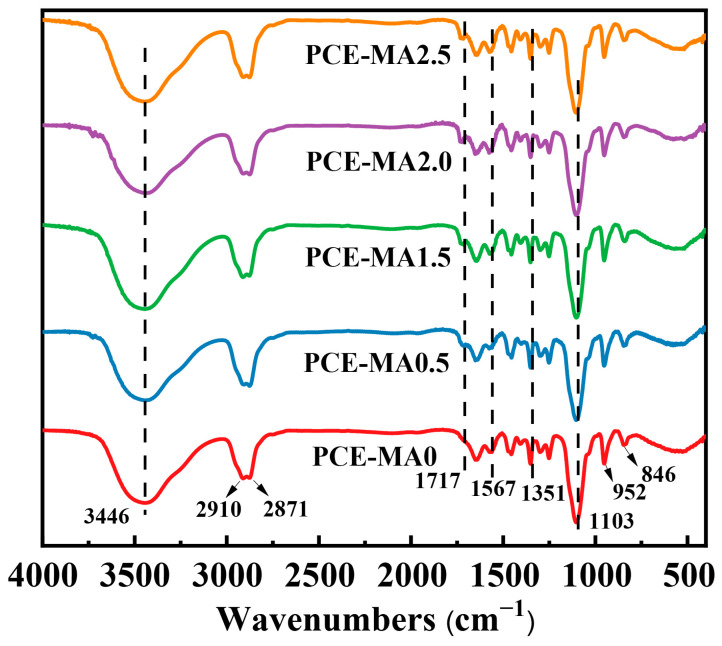
FT-IR spectra of the synthesized PCEs.

The characteristic groups and molecular structure of PCEs had been investigated by FT-IR spectrometer. Figure 3 presents the FT-IR spectrum curves of the synthesized PCEs.

For all the synthesized PCEs, a strong and blunt absorption band looms at around 3446 cm^−1^, implying the stretching vibration of -OH. The peaks at 2910 cm^−1^ and 2871 cm^−1^ attribute to the stretching vibration peaks of the C-H. The peaks at 956 cm^−1^ and 846 cm^−1^ refer to the out-of-plane bending vibration of -OH and -CH, respectively [51,52]. The stretching vibration peak of C-O-C in the HPEG molecular is at 1102 cm^−1^ [53]. The characteristic peak of sulfonic group (-SO_3_^−^) in SMAS appears around 1351 cm^−1^ [29,51], and the peak at 1567 cm^−1^ represents carboxyl group (-COO^−^) form AA [54]. The stretching vibration peak at 1717 cm^−1^ belongs to the ester group in MA, which presents in the curves of all the synthesized PCEs except PCE-MA0 [54,55]. All results validate the existence of characteristic groups in the PCEs molecules, aligning with our expectations.

#### 3.1.3. ^1^H NMR

**Figure 4:**
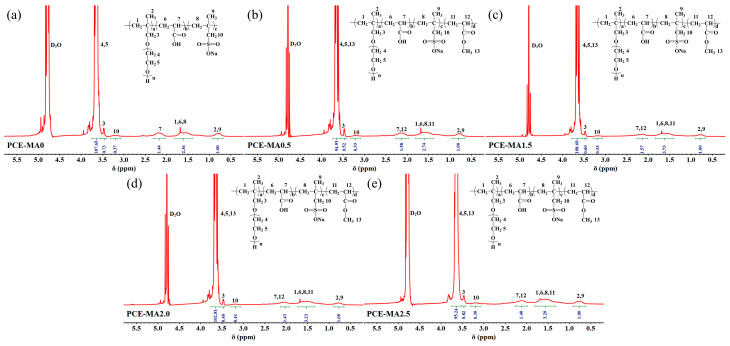
^1^H NMR spectrum of (**a**) PCE-MA0, (**b**) PCE-MA0.5, (**c**) PCE-MA1.5, (**d**) PCE-MA2.0, and (**e**) PCE-MA2.5.

^1^H NMR was used to further validate the introduction of the mentioned monomers on PCE molecules. The ^1^H NMR curves of PCEs are displayed in Figure 4.

From Figure 4, strong peak appears at around 4.70 ppm, which belongs to the solvent D_2_O [36]. In addition, the broad peaks at 0.67 ppm (H2 and H9) are ascribed to the H atoms of methyl groups directly attached to main chain. The peaks at around 1.46 ppm (H1, H6, H8, and H11) correspond to the -CH_2_- of backbones. Meanwhile, the peaks near 2.08 ppm (H7, H12) correspond to the H atom of tertiary carbon in the backbones. For the side chains of PCEs molecule, the strong and broad peaks (H4 and H5) near 3.54 ppm belong to the H atoms of -CH_2_CH_2_O- from HPEG [56]. Furthermore, the peak of H atoms in methyl group form MA appears around 3.6 ppm, which is covered by previous peaks. Meanwhile, the peak (H3) appears at 3.37 ppm attributed to -CH_2_^−^ from HPEG as well. The weak peaks (H10) at around 3.10 ppm stands for the H atom of methylene groups directly connected to S atom from SMAS. In addition, there is no obvious proton peak of CH_2_=CH_2_ in the range of 5.40–5.50 ppm, indicating the most of the C=C polymerized in the reaction [57]. These results confirm the successful polymerization of PCE molecules, aligning with the FT-IR data as exhibited in Figure 3.

### 3.2. Solution Conformation

**Figure 5:**
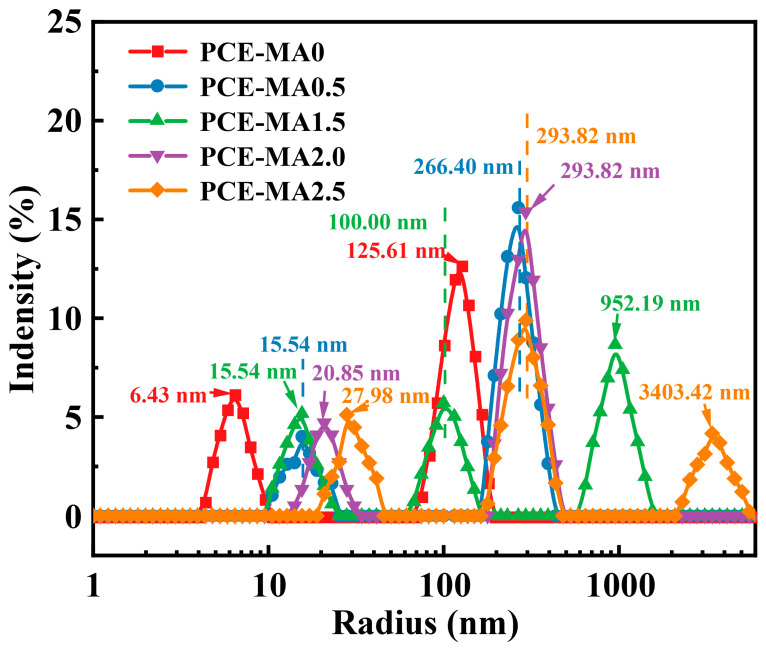
Hydrodynamic radius of synthesized PCEs.

To elucidate the influence of ester groups content on the conformation of PCEs molecules, the *R_h_* values of different PCEs in cement pore solutions were determined by DLS method, as displayed in Figure 5. In accordance with Figure 5, the dimension for PCE-MA0, PCE-MA0.5, PCE-MA1.5, PCE-MA2.0, and PCE-MA2.5 agglomerates is 125.61 nm, 266.40 nm, 952.19 nm, 293.82 nm, and 3403.42 nm, respectively [35,58]. The *R_h_* of single molecule of PCE-MA0, PCE-MA0.5, PCE-MA1.5, PCE-MA2.0, and PCE-MA2.5 in cement pore solutions is 6.43 nm, 15.54 nm, 15.54 nm, 20.85 nm, and 27.98 nm, respectively. Compared to other PCEs, the single molecule diameter of PCE-MA2.5 is larger, which can be attributed to its largest molecular weight [35]. In addition, compared with PCE-MA0, PCE-MA0.5 has larger *R_h_*, indicating the smaller conformational contraction of PCE-MA0.5 molecules and more exposure for anions group [59,60,61]. This deeply affects the difference in adsorption between PCE–MA0 and PCE-MA0.5.

### 3.3. Zeta Potential

**Figure 6:**
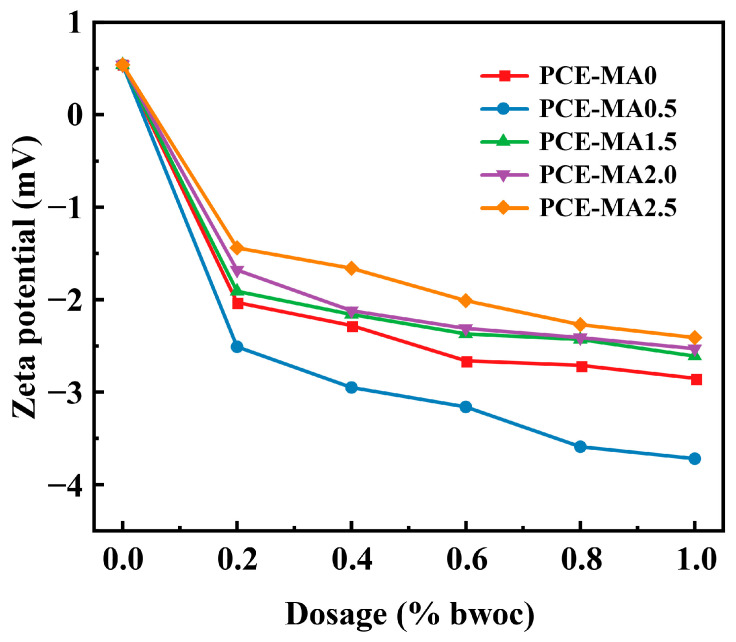
Zeta potential of cement slurry versus various PCEs dosage.

To explore the interplay between PCEs and cement, the zeta potential containing five different PCEs was measured. The results ranged from 0% to 1% (bwoc) presents in Figure 6.

As illustrated in Figure 6, it is obvious that once PCEs were added, the zeta potential rapidly decreased from a positive value (+0.53 mV) to negative. This is because the adsorption of positively charged cement particles by anionic groups in PCEs molecules, which forms a double electric layer and converts zeta potential to negative [62,63]. And with the escalation of PCEs dosage, the value of zeta potential of all samples declines, and the decrease rate of zeta potential gradually diminishes because the adsorption amount of PCEs on the cement particles tend to be saturated [64,65]. In addition, due to the greater rigidity of the PCE-MA0.5 backbone, PCE-MA0.5 exposes more anionic groups, leading to a lower zeta potential value of PCE-MA0.5 than PCE-MA0 which theoretically possesses a higher anion density. Meanwhile, for all PCEs containing ester groups, with the increase of the ester groups in the backbone, the anion density decreases, manifested by the increase of zeta potential value. Compared with other PCEs containing ester groups, PCE-MA0.5 has the least ester groups and exhibits the lowest zeta potential at all dosages, indicatingPCE-MA0.5 has the strongest adsorption capacity.

### 3.4. Surface Tension

**Figure 7:**
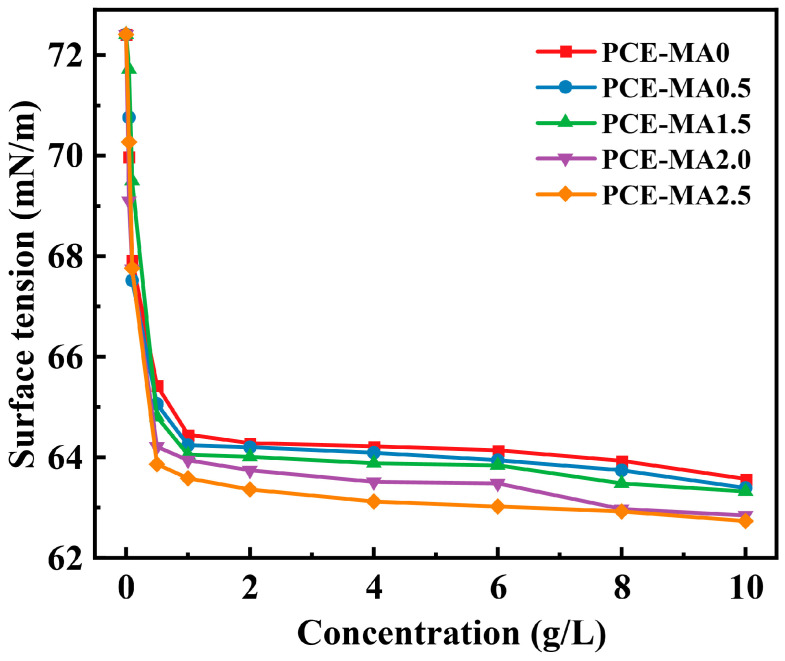
Surface tension of PCE solutions versus PCE concentration.

The relationship between ester group content of PCEs molecular and surface tension was examined, as demonstrated in Figure 7. With the escalating consistence of PCEs solution, the surface tension of the PCEs solution diminishes from surface tension of DI water (72.41 mN/m). At the same dosage, the higher the ester groups content in the PCEs molecule, the less the surface tension of the solutions. This phenomenon can be ascribed to the introduction of hydrophobic groups (methyl groups) resulting to the decrease of the interaction between PCEs and water, thus releasing some free water, which is beneficial to reduce the viscosity of paste [29,66].

### 3.5. Adsorption Amount of PCEs on Cement

**Figure 8:**
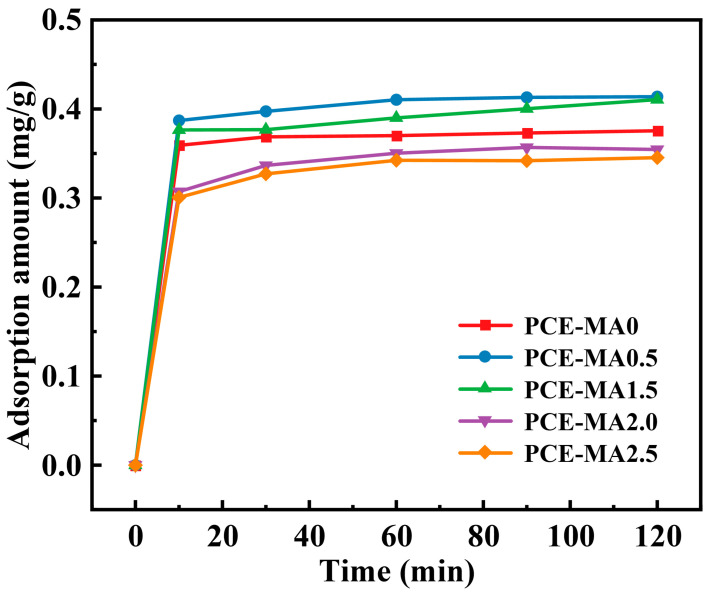
Variation of PCE adsorption on cement within 2 h.

The adsorption capacity stems from PCE molecular structure, in particular the density of anionic groups in the backbones of PCEs. PCE molecular can bind to cement surfaces by chelating with Ca^2+^ on cement particles through anionic groups on the backbones as anchors [67]. In addition, it has been reported that the fluidity of cement paste grows as the increasing amount of adsorption of PCEs on its particles [68]. Thus, to investigate the effect of PCEs on cement, variation in the adsorption amount of synthesized PCEs on cement particles within 2 h was gauged, resulting in Figure 8.

As illustrated Figure 8, the adsorption amount of all synthesized PCEs rapidly increases and the rates of adsorption maximize within 10 min. After that, the adsorption amount of all PCEs is gradually growing except for PCE-MA0. At 30 min, PCE-MA0 adsorption amount reaches a plateau and adsorption rate decreases to zero. This can be attributed to no ester group introduced in the PCE-MA0 molecules. In the alkaline environment of cement, ester groups continuously hydrolyze to produce new -COO^−^ to adsorb cement particles [68,69]. The adsorption groups including carboxyl groups and sulfonic group of PCE-MA0 are rapidly consumed within 30 min. The result proves the occurrence of ester hydrolysis within 2 h, and aligns with the results of the fluidity retention test. Besides, PCE-MA0.5 has a higher adsorption amount than PCE-MA0 due to the fact that the backbone of PCE-MA0.5 molecular shrinks less in alkaline environment leading to PCE-MA0.5 molecular exposes more anions.

**Figure 9:**
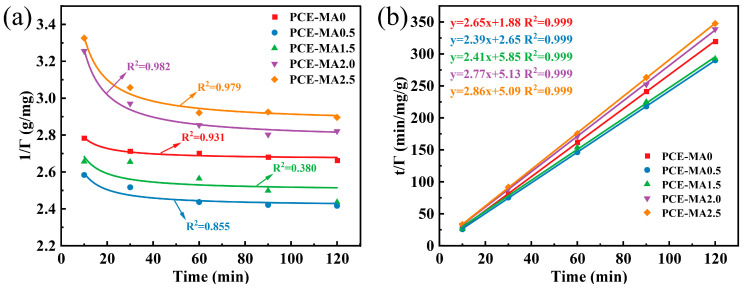
Adsorption kinetics of PCEs on the cement (**a**) Pseudo-first-order model and (**b**) Pseudo-second-order model.

Adsorption kinetics of cement particles with PCEs was suitable for pseudo-first-order and pseudo-second-order kinetic equations, presented in Figure 9 and Table 5. The saturated adsorption capacities of PCE-MA0, PCE-MA0.5, PCE-MA1.5, PCE-MA2.0, and PCE-MA2.5 calculated by the pseudo-second-order model (0.377, 0.421, 0.414, 0.361, and 0.350 mg/g, respectively) were found to be close to the experimental values in Figure 9. In addition, the R2 values of these fitting curve of the pseudo-second-order model are all 0.999. The findings verify the pseudo second order model for adsorption kinetics of these five synthetic PCEs, indicating chemical adsorption dominant in the adsorption process.

### 3.6. Fluidity and Fluidity Retention Abilities

**Figure 10:**
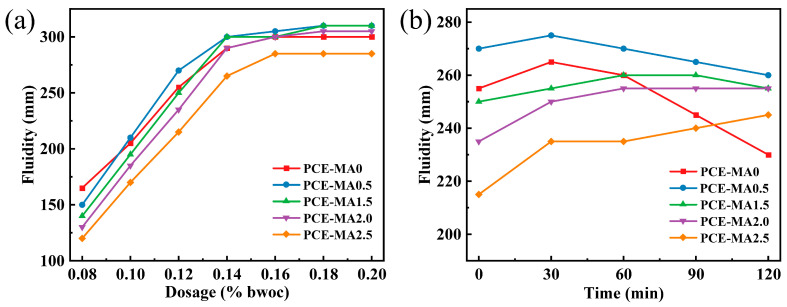
(**a**) Initial fluidity and (**b**) fluidity within 2 h of cement slurry with all PCEs.

Figure 10 shows the initial fluidity of cement slurry across varying dosages ranged from 0.08% to 2.0% (bwoc) of all the synthesized PCEs. From Figure 10, the initial fluidity of cement slurry boosts with escalating PCE dosages until reaching the plateau, and the height of plateau can be used for evaluating the dispersion ability of PCEs. The fluidity of PCE-MA0.5 is the highest for almost every dosage, indicating PCE-MA0.5 has the best dispersion ability. The lower fluidity of PCE-MA0 compared to PCE-MA0.5 is due to the more pronounced conformational contraction of PCE-MA0 molecular, which results in the cover of anionic groups on its main chain and leads to lower adsorption amount. For PCE-MA0.5 to PCE-MA2.5, the initial fluidity of cement paste displays a diminishing trend as the proportion of MA in PCE increases. This negative trend can be related to the increase of MA leading to relative reduction of the proportion pf AA and SMAS in PCEs molecular, resulting in the reduction of the adsorption amount [66,70,71,72]. Therefore, PCE-MA0.5 exhibits superior dispersion ability.

In the practical application of PCEs, it is necessary to ensure the workability of cement paste for a period of time, so evaluating the fluidity retention abilities of cement slurry is crucial. Thus, the fluidity of cement slurry incorporating 0.12% bwoc PCEs within 2 h has been measured, resulting in Figure 10b. From Figure 10b, the fluidity of cement paste containing PCE-MA0 arrives its maximum value of 265 mm at 30 min, and its fluidity drops sharply to 230 mm in the following 90 min. Compared with other samples, it can be seen that this decrease gradually fades with the increase of MA content in the molecular, and PCE-MA2.5 with the highest MA content even maintains growth in the fluidity of cement paste within 120 min. The phenomenon results from the ester groups in MA, which continuously hydrolyze into -COO^−^ in the alkaline environment and adsorb onto the surface of cement particles [31,55,73]. Additionally, note that PCE-MA0.5, despite deceasing a little of its fluidity within 2 h, maintained good fluidity for 2 h due to its high initial fluidity. Compared with PCE-MA0.5, PCE-MA0 without MA in the molecular not only has lower initial fluidity, but also decreases 9.8% fluidity within 2 h. These results have proven that introducing a small amount of MA into the PCEs molecule contributes to have excellent fluidity and suppress fluidity loss.

### 3.7. Hydration Heat

**Figure 11:**
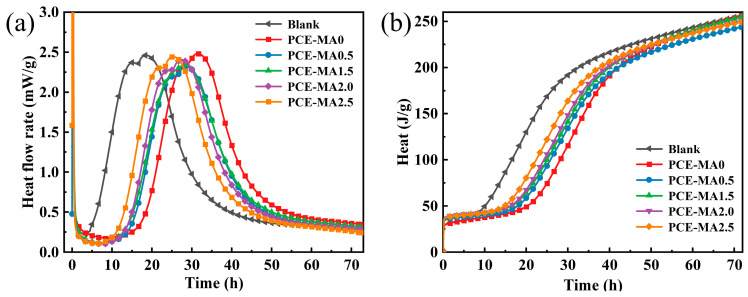
Hydration heat of cement pastes with various PCEs (**a**) heat flow and (**b**) hydration heat.

The impact of synthetic PCEs containing different amounts MA on the hydration of cement was probed through isothermal heat flow calorimetry. Figure 11 exhibits the exothermic curves of hydration for blank cement slurry and cement slurries incorporating various PCEs within 72 h.

In contrast with blank cement paste without PCEs, cement slurry incorporating various PCEs encountered notable delays in hydration. This is attributed to the absorption of PCEs on the cement surface, forming an enclosing layer restricting the interface exchange of water and ions, thereby impairing cement hydration [55,74]. Further, the heat of hydration curves of cement for PCE with MA molecules are all to the left compared to PCE without ester groups, implying that the introduction of MA molecules does not delay hydration, which aligns with the conclusions drawn by Kong et al. [75]. Compared to PCE containing MA molecules, PCE-MA0 has a higher heat flow peak and hydration heat. During hydration, adsorbed groups are continually depleted and desorbed. In the alkaline environment of the cement, PCEs containing ester groups hydrolyze and generate adsorption groups (-COO^−^), thereby inhibiting the dramatic increase of heat flow [75]. This also explains why PCE-MA0 has a higher peak heat flow and hydration heat compared to PCE containing MA molecules. In addition, hydration degree can be partially mirrored in the heat generated by cement paste [75]. From Figure 11b, after 72 h of cement hydration, the cement paste containing PCE-MA0.5 released the least amount of heat, demonstrating the lowest degree of hydration, which can be attributed to the strongest adsorption capacity of PCE-MA0.5, thus reducing the degree of hydration [76]. This is also supported by the adsorption results (Figure 8).

### 3.8. Setting Time

**Figure 12:**
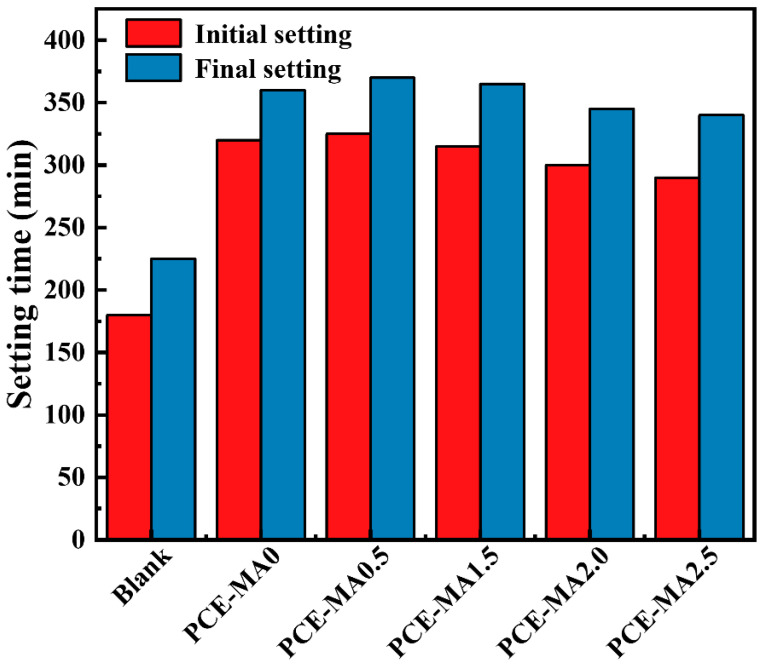
The initial and final setting time of PCEs at a dosage of 1.20% (w/c = 0.27).

The initial setting time and the final setting time of the blank sample and the samples containing PCEs are shown in the Figure 12. It can be seen from the diagram that once PCEs are added to the cement paste, the setting time increases significantly. This can be attributed to the adsorption of PCEs on the cement surface, forming a coating layer that limits the exchange of water and cement particles, thereby damaging cement hydration and delaying setting time of cement [55]. In addition, for all cement pastes containing PCEs, the setting time decreases with the increase of PCEs ester content. In the alkaline environment of cement, the ester group will hydrolyze to produce adsorption groups, which will damage the hydration of cement and prolong the setting time [77]. However, when the proportion of MA increases, the density of PCE side chains will be greatly reduced, resulting in a decrease in the steric hindrance of PCE and a thinning of the adsorption layer on the surface of cement particles. Therefore, cement particles are expected to have more opportunities to contact with water, thus weakening the delayed effect of PCE on cement hydration and shortening the setting time [66,71,78].

### 3.9. WFT Results

**Figure 13:**
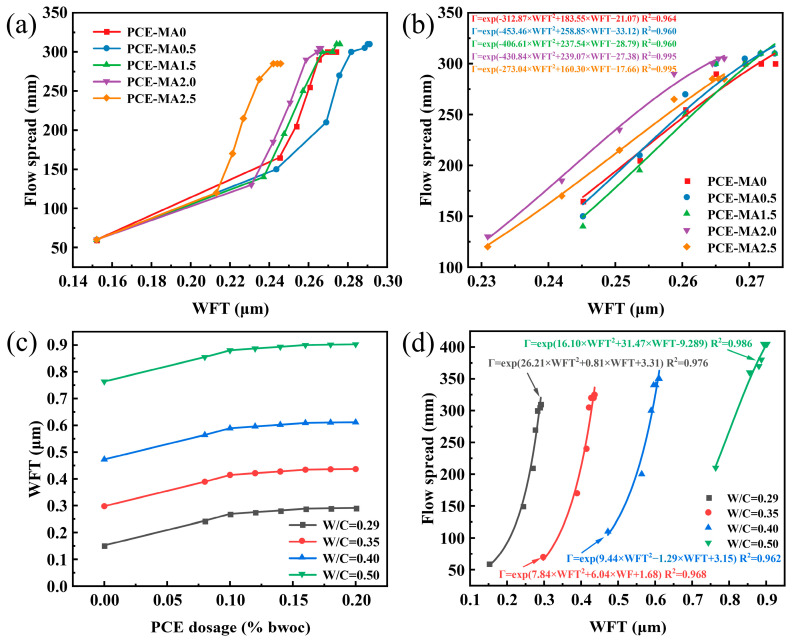
WFT of (**a**) different PCEs versus dosages and (**b**) fluidity versus WFT. WFT for PCE-MA0.5 of (**c**) different w/c ratios versus dosages and (**d**) fluidity versus WFT. The solid lines in (**b**,**d**) depict the fitting outcomes.

The influence of various PCEs on WFT was evaluated via the calculation of WFT from cement pastes with w/c of 0.29, resulting in Figure 13a and Table S1. For all PCEs, WFT increased and eventually levelled off with increasing PCE content, indicating that the adsorption of PCEs on cement particles reaches saturation [79]. PCE inclusion enhanced cement packing density, consequently impacting excess water volumes. The larger the dosage of PCEs is, the larger the excess water volume is. In addition, evaluating WFT curves for diverse PCEs reveals that PCE-MA0.5 displays higher WFT values across nearly all dosages. For the PCE-MA0.5 curve, there is a 79.9% increase in WFT with the dosage increased from 0% to 2.0%, as a comparison for the PCE-MA2.5 curve, that is 61.5%. Revealingly, PCE-MA0.5 outperforms other PCEs when boosting WFT as it enhances packing density more effectively. This phenomenon may be related to the high adsorption amount of PCE-MA0.5, which is consistent with the results of Ren et al. [80].

To comprehend how varying w/c impact the WFT, the WFT of cement pastes containing PCE-MA0.5 with various w/c ratios was measured. These results are illustrated in Figure 13b and detailed in Table S1. The WFT of cement pastes containing PCE-MA0.5 increases from 0.15 μm to 0.27 μm at 0.29 w/c, an increase of 79.9%, while at 0.50 w/c, the WFT increases from 0.76 μm to 0.90 μm, an increase of 18.2%. Therefore, with the increase of PCE-MA0.5 dosages, the increase of WFT at lower w/c ratio is more obvious than higher w/c ratio. This is due to the fact that at high w/c ratios, the mixed water is sufficient to fill the voids, thus reducing effect of PCEs, which aligns with the findings of prior study [79,81].

To investigate the correlation between WFT and fluidity, WFT and fluidity data for distinct PCEs and varying w/c ratios were analyzed, as shown in Figure 13b and Figure 13d, respectively. The employed fitting equation aligns with Zhao et al. [82]. From Figure 13b, the R^2^ values are greater than 0.960. In Figure 13d, with the same w/c, as WFT augments, fluidity following suits. In addition, with the same dosage of the same PCE, all w/c ratios exhibit high consistency (R^2^ > 0.962). The above results indicate that WFT and fluidity show a high correlation whether it is different PCE or the same PCE with different w/c ratios. This can be attributed to augmenting WFT reducing the friction and cohesiveness between particles between solid particulates, thereby enhancing pastes fluidity, corroborating prior findings [79,82,83].

### 3.10. Rheological Behavior of Cement Paste

**Figure 14:**
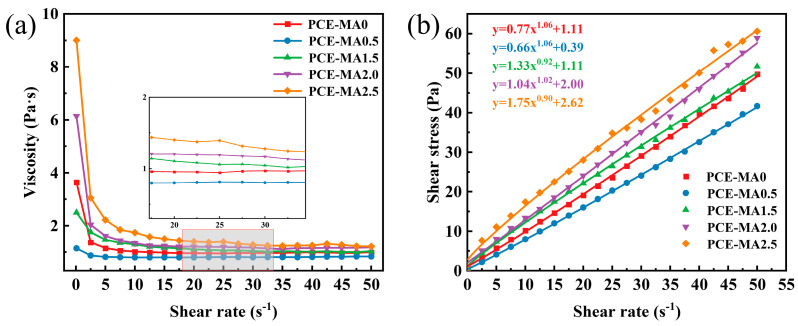
Rheological properties of cement pastes containing various PCEs, (**a**) the apparent viscosity and (**b**) the shear stress. The solid lines in (**b**) represent the fitting results.

Rheological parameters embodying yield stress and plastic viscosity are important criteria for evaluating the fluidity of cement paste [36]. The rheological properties of cement paste containing synthesized PCEs, including shear stress and apparent viscosity, were examined with 0.29 w/c and 0.12% bwoc, as illustrated in Figure 14. After that, the rheological properties were assessed via the Herschel-Bulkley model and the fitting outcomes are displayed in Figure 14 and Table 6.

As presented in Figure 14, PCE-MA0.5 exhibits the best rheological properties, i.e., the lowest shear stress and plastic viscosity at identical shear rate. This is causally linked to its stronger adsorption behavior leading to superior dispersion properties, reducing the cohesive forces between cement particles [84,85]. From Herschel-Bulkley model rheological parameters of PCE-MA0.5, the rheological index (n) is over 1 and the consistency coefficient (K) is the lowest, demonstrating that its cement paste is dilatant fluid with lowest viscosity [84]. It has been reported that the greater the adsorption amount and thickness of PCEs, the lower the yield stress and plastic viscosity of cement paste containing the corresponding PCEs [69,86]. Compared with PCE-MA0.5, PCE-MA0 without ester group has higher viscosity and shear stress due to lower in adsorption amount. In addition, with escalating of the ester content in the PCEs molecule, the viscosity and shear stress of the corresponding cement paste grow. The findings align succinctly with the fluidity results.

### 3.11. Mortar Strength

**Figure 15:**
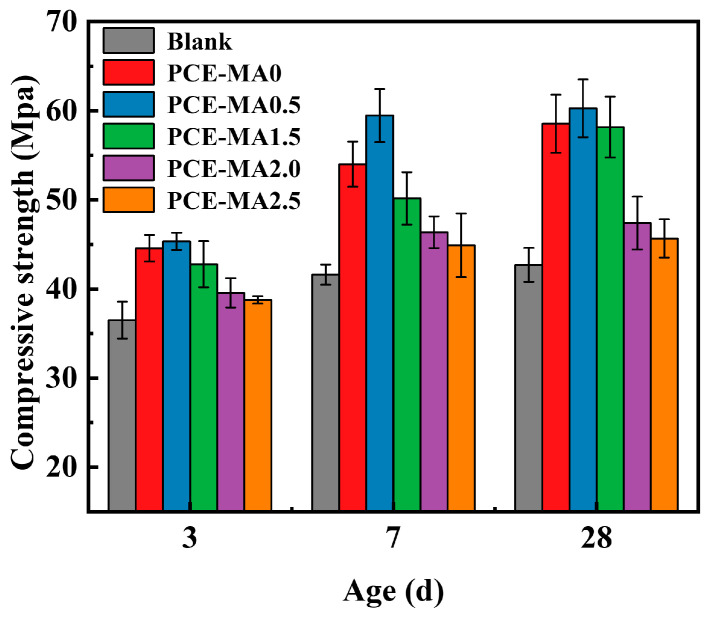
Compressive strength of cement mortar incorporating diverse PCEs.

As illustrated in Figure 15, the influence of synthesized PCEs on the compressive strength of mortar over varied curing durations was investigated. The diagram exhibits that the synthesized PCEs augmented the compressive strength. In comparison with blank samples without PCEs, once PCEs were mixed with mortar, the compressive strength of mortar escalated post 3, 7 and 28 d. The mortar blocks containing PCE-MA0.5 presented the greatest compressive strength on account of its superior dispersion, due to its excellent dispersibility, which makes the distribution of pores in the sample more uniform in distribution and size [50,87,88].

### 3.12. Performances of the PCEs in Concrete

To assess the viscosity reduction property of the synthesized PCEs, rheological analysis on fresh concrete incorporating various PCEs was conducted, listed in Table 7 and Table S2.

As exhibited in Table 7, by adjusting to the dosage of PCEs, the slump flow of concretes was control in the range of 600 ± 20 mm. In previous studies, T_500_ and efflux times were used to evaluate the viscosity of concrete [89]. From Table 7, as the content of ester groups in PCEs increases, T_500_ and efflux time decrease under similar conditions of slump, slump flow, and air content. This can be ascribed to introduce hydrophobic ester groups into the PCEs molecular reduce the interfacial tension between cement particles and water, which can effectively improve the viscosity reduction effect of PCEs [29]. This is consistent with the discussion in surface tension. In addition, it can be seen that the T_500_ and efflux time of concrete with PCE-MA2.5 are as short as 2.17 s and 3.53 s respectively, but its dosage is relatively high (1.25%). This may be because its highest ester content reduces its anionic density, leading to weaker adsorption capacity on cement particles, corresponding to the adsorption results above. For PCE-MA0.5, although its T_500_ and efflux time are not lowest, its high adsorption ensures that its dosage is far lower than the other PCEs when its slump flow reaching 600 ± 20 mm. Therefore, the synthesized PCE-MA0.5 presents remarkable potential for concrete applications that require low viscosity and superior fluidity.

### 3.13. Working Mechanism of the PCEs

**Figure 16:**
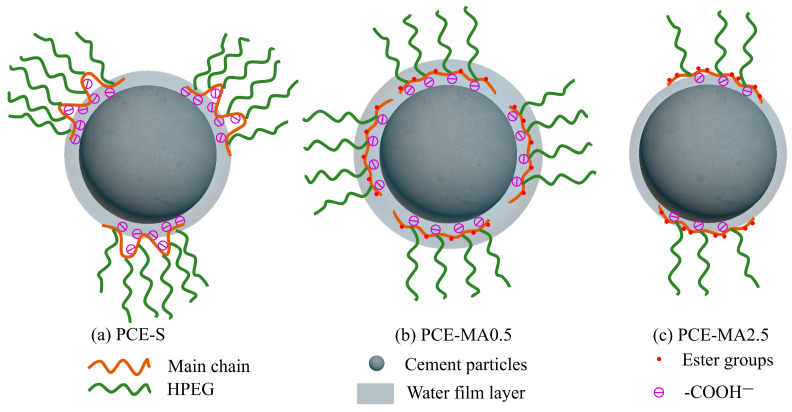
The working mechanism diagram of PCEs.

In light of aforementioned analysis results, the working mechanism for the PCEs was revealed and displayed in Figure 16. By contrast, PCE-MA0.5 possesses prime application potential. Firstly, the presence of an ester group in the PCEs molecule of PCE-MA0.5 gives it a lower loss of fluidity over 2 h. Compared to PCE-MA0 containing highest theoretical anion density, the introduction of ester groups results in PCE-MA0.5 having a more rigid backbone, thus exposing more anions on its backbone. Meanwhile, compared to the rest of PCEs containing more ester groups, PCE-MA0.5 has higher anion density. As a result, PCE-MA0.5 exhibits the strongest adsorption capacity, leading to the thickest water film layer and the best fluidity. In addition, the existence of the hydrophobic MA groups helps to reduce the surface tension of PCEs aqueous solutions, as displayed in surface tension results. The hydrophobic ester group greatly reduces the force between water and cement particles, thus achieves viscosity-reducing effect in the concrete. Form concrete test, the more ester groups in the PCEs solutions, the shorter of efflux time and T_500_ of the concrete. However, the lower adsorption capacity of PCE with high content of ester groups also needs to be taken into account.

## 4. Conclusions

An array of viscosity-reducing PCEs with different ester group contents were synthesized using HPEG, AA, MA and SMAS as the reaction monomers. The synthesized samples were sequentially subjected to FTIR, ^1^H MMR, GPC, and DLS confirmed the designed molecular structure. The influence on zeta potential, surface tension, adsorption, rheological properties, fluidity, hydration heat, WFT, compressive strength, and workability of concrete was investigated.

The synthesized PCE-MA0.5 exhibited the highest adsorption capacity, the lowest zeta potential, and the best flowability. This superiority is also seen in rheological testing and concrete property testing. In rheological tests, cement pastes containing PCE-MA0.5 has the lowest viscosity; in concrete tests, it was the lowest dosage for the similar slump flow.

In this paper, it was found that introducing a small amount of MA into the backbones helps to improve its rigidity to expose more anion groups, leading to higher adsorption amount and better fluidity. PCE-MA0.5 exhibited the highest adsorption capacity and the best flowability. As the ester group content increases further, the adsorption capacity of the synthesized PCEs decreases due to the decrease of anion density on backbones. Meanwhile, it is worth noting from the surface tension that the higher the content of hydrophobic ester groups in the molecule, the lower surface tension. The results of T_500_ and efflux time of concrete indicate that the increase of ester groups content in PCEs can effectively enhance the viscosity reduction effect due to the decease of force between water and cement particles. At the same time, it should also be considered that as the increase of ester group content, adsorption capacity decreases, leading to an increasing dosage of PCE in concrete.

This research aims to provide an effective approach for enhancing dispersion effect and reducing viscosity utilizing our innovative molecular design. This study presents the inaugural evaluation of augmenting contents of ester groups in the presence of sulfonic acid groups to the workability of the synthetic PCEs in freshly poured concrete. The significance of this research is to outline an effective approach to produce an innovative, viscosity reducing PCEs with high potential in concrete applications.

In this paper, only the influence of polycarboxylate superplasticizer containing MA on the performance of concrete under normal conditions is studied, and the influence of other ester-containing groups (such as hydroxyl ethyl acrylate, and 2-hydroxypropyl acrylate) should be further studied. Moreover, in the in practical application, the long-term durability of concrete will face the challenge of harsh environment (such as high altitude, cold, and ultra-high temperature). The hydrolysis of ester groups is also affected by environmental factors (such as temperature, humidity, and pH). Therefore, it is necessary to carry out in-depth research on the hydrolysis process of ester-containing PCEs in concrete under harsh environment in the future. And by studying the effect of ester hydrolysis on the long-term tolerance of concrete in harsh environments, it will provide a reference for the PCEs industry.

## Figures and Tables

**Table 1:** Chemical and mineral components of reference cement.

Chemical Composition	wt.%	Mineral Composition	wt.%
SiO_2_	20.4	C_3_S	58.94
Al_2_O_3_	4.40	C_2_S	15.31
Fe_2_O_3_	3.27	C_3_A	6.71
CaO	62.70	C_4_AF	11.58
MgO	2.86		
SO_3_	2.18		
Na_2_Oeq	0.59		
f-CaO	0.78		
Loss	1.75		
Cl^−^	0.018		

**Table 2:** Monomers molar ratio of PCEs.

Sample	HPEG (a)	AA (b)	SMAS (c)	MA (d)
PCE-MA0	1	4	0.04	0
PCE-MA0.5	1	4	0.04	0.5
PCE-MA1.5	1	4	0.04	1.5
PCE-MA2.0	1	4	0.04	2.0
PCE-MA2.5	1	4	0.04	2.5

**Table 3:** Mix proportion of concrete (kg/m^3^).

Components	Cement	Sand	Gravel		Water
			(5–10 mm)	(10–20 mm)	
Mass (kg/m^3^)	500	808	182	730	180

**Table 4:** Characterization of various PCEs molecules.

Sample	M_n_	M_w_		PDI (M_w_/M_n_)	*R*_*h*_ (%)
		Peak1	Peak2		
PCE-MA0	23,700	47,281	2052	1.99	81.9
PCE-MA0.5	22,929	48,992	2035	2.14	87.8
PCE-MA1.5	24,524	57,841	1950	2.36	89.3
PCE-MA2.0	24,926	55,174	1998	2.21	87.8
PCE-MA2.5	25,353	74,872	2026	2.95	92.4

**Table 5:** Adsorption kinetic parameters of PCEs on the cement.

Samples	Pseudo-First-Order Model			Pseudo-Second-Order Model		
	*K*_1_	*Γ*_∞_	*R* ^2^	*K*_2_	*Γ*_∞_	*R* ^2^
PCE-MA0	0.443	0.375	0.931	3.755	0.377	0.999
PCE-MA0.5	0.749	0.414	0.855	2.928	0.421	0.999
PCE-MA1.5	0.740	0.400	0.380	0.995	0.414	0.999
PCE-MA2.0	1.758	0.360	0.982	1.492	0.361	0.999
PCE-MA2.5	1.628	0.349	0.979	1.604	0.350	0.999

**Table 6:** Herschel-Bulkley model rheological parameters.

Model	Sample	Formula	Parameter				
			*τ*_0_/Pa	*K*/Pa·s^n^	*n*	*μ*/Pa⋅s	*R* ^2^
Herschel-Bulkley model	PCE-MA0	*τ* = *τ*_0_ + *K*γ^*n*^	1.11	0.77	1.06	0.95	0.999
	PCE-MA0.5		0.39	0.66	1.06	0.82	0.997
	PCE-MA1.5		1.11	1.32	0.92	0.99	0.998
	PCE-MA2.0		2.00	1.04	1.02	1.12	0.998
	PCE-MA2.5		2.62	1.75	0.9	1.22	0.995

**Table 7:** Rheological performance of concrete containing various PCEs.

Sample	Dosage (%)	Slump/Slump Flow (mm)	T_500_ (s)	Efflux Time (s)	Air Content (%)
PCE-MA0	0.85	240/590	9.85	7.68	2.3
PCE-MA0.5	0.80	240/620	7.31	7.13	2.4
PCE-MA1.5	1.00	230/615	4.76	6.20	2.2
PCE-MA2.0	1.05	235/620	3.68	5.15	1.9
PCE-MA2.5	1.25	240/615	2.17	3.53	2.1

## Data Availability

The original contributions presented in the study are included in the article.

## Supplementary Materials

The following supporting information can be downloaded at: https://www.mdpi.com/article/10.3390/polym16233272/s1, Table S1: Packing Density and WFT results of paste mixes; Table S2: Rheological properties of all concrete mixtures.
