# Supplementary material for: Effect of Density of Acrylic Acid Ester on Sulfonate-Modified Polycarboxylate Superplasticizers on Cementitious Systems
Source: Polymers (Basel). 2024 Nov 24;16(23):3272. doi: 10.3390/polym16233272 (PMC11644611; doi:10.3390/polym16233272)
Supplement: Supplementary file 1 [file polymers-16-03272-s001.zip › polymers-3269161-supplementary.pdf]

Supplementary Materials for

Effect of density of acrylic acid ester on sulfonate-modified polycarboxylate superplasticizers on cementitious systems

Yuxiang Xie[a], Zixuan Zhang[b], Yujie Chen[a], Xu Ren[a,c], Yuan Liu[d,e], Jia Tao[d,e], Runxia Liu[d,e], Min Li[a,c,], Ziwei Li[a,*]

[a]Guizhou Provincial Key Laboratory of Green Chemical and Clean Energy Technology, School of Chemistry and Chemical Engineering, Guizhou University, Guiyang 550025 (China);

[b]North Alabama International College of Engineering and Technology, Guizhou University, Guiyang 550025 (China);

[c]School of Civil Engineering, Guizhou Institute of Technology, Guiyang 550025 (China);

[d]China Railway Fifth Bureau Group Co., LTD, Guiyang 550003 (China);

[e]Guizhou Tianwei Building Materials Technology Co., LTD, Guiyang 550025 (China).

*** Corresponding authors:**

Ziwei Li, Email: zwli@gzu.edu.cn

1. WFT results.

Table S1. Packing Density and WFT results of paste mixes.

Mix No.	Packing Density	Excess water ratio	Specific surface area (m^2 /m^3)	WFT (μm)
Blank-0-0.29	0.575	0.163	1068000	0.152
MA0-0.08-0.29	0.610	0.262	1068000	0.245
MA0-0.10-0.29	0.613	0.271	1068000	0.254
MA0-0.12-0.29	0.616	0.278	1068000	0.260
MA0-0.14-0.29	0.618	0.283	1068000	0.265
MA0-0.16-0.29	0.619	0.288	1068000	0.269
MA0-0.18-0.29	0.620	0.290	1068000	0.272
MA0-0.20-0.29	0.621	0.292	1068000	0.274
MA0.5-0.08-0.29	0.609	0.260	1068000	0.244
MA0.5-0.10-0.29	0.619	0.287	1068000	0.269
MA0.5-0.12-0.29	0.622	0.294	1068000	0.276
MA0.5-0.14-0.29	0.625	0.301	1068000	0.282
MA0.5-0.16-0.29	0.627	0.308	1068000	0.289
MA0.5-0.18-0.29	0.628	0.310	1068000	0.290
MA0.5-0.20-0.29	0.629	0.311	1068000	0.291
MA1.5-0.08-0.29	0.607	0.253	1068000	0.237

MA1.5-0.10-0.29	0.611	0.264	1068000	0.248
MA1.5-0.12-0.29	0.615	0.275	1068000	0.257
MA1.5-0.14-0.29	0.618	0.285	1068000	0.267
MA1.5-0.16-0.29	0.621	0.291	1068000	0.272
MA1.5-0.18-0.29	0.622	0.293	1068000	0.274
MA1.5-0.20-0.29	0.622	0.295	1068000	0.276
MA2.0-0.08-0.29	0.604	0.247	1068000	0.231
MA2.0-0.10-0.29	0.608	0.258	1068000	0.242
MA2.0-0.12-0.29	0.612	0.268	1068000	0.251
MA2.0-0.14-0.29	0.615	0.276	1068000	0.259
MA2.0-0.16-0.29	0.618	0.282	1068000	0.264
MA2.0-0.18-0.29	0.618	0.283	1068000	0.265
MA2.0-0.20-0.29	0.618	0.284	1068000	0.266
MA2.5-0.08-0.29	0.597	0.228	1068000	0.213
MA2.5-0.10-0.29	0.600	0.236	1068000	0.221
MA2.5-0.12-0.29	0.603	0.242	1068000	0.227
MA2.5-0.14-0.29	0.606	0.251	1068000	0.235
MA2.5-0.16-0.29	0.609	0.259	1068000	0.242
MA2.5-0.18-0.29	0.609	0.261	1068000	0.244
MA2.5-0.20-0.29	0.610	0.262	1068000	0.246

Blank-0-0.35	0.575	0.318	1068000	0.298
MA0.5-0.08-0.35	0.609	0.416	1068000	0.389
MA0.5-0.10-0.35	0.619	0.443	1068000	0.415
MA0.5-0.12-0.35	0.622	0.450	1068000	0.421
MA0.5-0.14-0.35	0.625	0.456	1068000	0.427
MA0.5-0.16-0.35	0.627	0.464	1068000	0.434
MA0.5-0.18-0.35	0.628	0.465	1068000	0.436
MA0.5-0.20-0.35	0.629	0.466	1068000	0.437
Blank-0-0.40	0.575	0.505	1068000	0.472
MA0.5-0.08-0.40	0.609	0.602	1068000	0.564
MA0.5-0.10-0.40	0.619	0.629	1068000	0.589
MA0.5-0.12-0.40	0.622	0.637	1068000	0.596
MA0.5-0.14-0.40	0.625	0.643	1068000	0.602
MA0.5-0.16-0.40	0.627	0.650	1068000	0.609
MA0.5-0.18-0.40	0.628	0.652	1068000	0.610
MA0.5-0.20-0.40	0.629	0.653	1068000	0.611
Blank-0-0.50	0.575	0.816	1068000	0.764
MA0.5-0.08-0.50	0.609	0.913	1068000	0.855
MA0.5-0.10-0.50	0.619	0.940	1068000	0.881
MA0.5-0.12-0.50	0.622	0.948	1068000	0.877
MA0.5-0.14-0.50	0.625	0.954	1068000	0.893

MA0.5-0.16-0.50	0.627	0.961	1068000	0.900
MA0.5-0.18-0.50	0.628	0.963	1068000	0.902
MA0.5-0.20-0.50	0.629	0.964	1068000	0.903

Note: In the sample number X-Y-Z, X is short for the name of various PCEs, like MA0 is short for PCE- MA0, and Blank means no PCEs in pastes; Y denotes PCEs dosages (bwoc); and Z denotes w/c ratios.

2. Performances of the PCEs in concrete.

Table S2. Rheological properties of all concrete mixtures.

Sample	Dosage (%)	Slump/slump flow (mm)	T_{500} (s)	Efflux time (s)	Air content (%)
PCE-MA0	0.80	220/470	-	11.18	2.1
PCE-MA0	0.85	240/590	9.85	7.68	2.3
PCE-MA0	0.90	245/690	6.35	6.19	2.4
PCE-MA0.5	0.75	225/500	9.63	10.08	2.1
PCE-MA0.5	0.80	240/620	7.31	7.13	2.4
PCE-MA0.5	0.85	250/700	4.58	5.60	2.4
PCE-MA1.5	0.95	225/510	8.36	9.35	2.2
PCE-MA1.5	1.00	230/615	4.76	6.20	2.2
PCE-MA1.5	1.05	240/680	3.45	5.03	1.8
PCE-MA2.0	1.00	225/480	-	6.51	1.8
PCE-MA2.0	1.05	235/620	3.68	5.15	1.9
PCE-MA2.0	1.10	240/710	2.12	3.05	2.2
PCE-MA2.5	1.20	230/500	7.89	6.04	1.7
PCE-MA2.5	1.25	240/615	2.17	3.53	2.1

PCE-MA2.5	1.30	240/720	2.03	2.85	1.9
